# Diversity of Non-O157 Shiga Toxin-Producing *Escherichia coli* Isolated from Cattle from Central and Southern Chile

**DOI:** 10.3390/ani11082388

**Published:** 2021-08-13

**Authors:** Leonela Díaz, Sebastian Gutierrez, Andrea I Moreno-Switt, Luis Pablo Hervé, Christopher Hamilton-West, Nora Lía Padola, Paola Navarrete, Angélica Reyes-Jara, Jianghong Meng, Narjol González-Escalona, Magaly Toro

**Affiliations:** 1 Instituto de Nutrición y Tecnología de los Alimentos (INTA), Universidad de Chile, Macul 7830490, Santiago, Chile; leoneladiaz@gmail.com (L.D.); sebastiangutierrez@ug.uchile.cl (S.G.); pnavarre@inta.uchile.cl (P.N.); areyes@inta.uchile.cl (A.R.-J.); 2 Escuela de Medicina Veterinaria, Facultad de Agronomía e Ingeniería Forestal, Facultad de Ciencias Biológicas, Facultad de Medicina, Pontificia Universidad Católica de Chile, Macul 7820244, Santiago, Chile; andrea.moreno@uc.cl; 3 ANID—Millennium Initiative for Collaborative Research on Bacterial Resistance (MICROB-R), Avenida Las Condes 12461, Lo Barnechea 7690000, Santiago, Chile; 4 Facultad de Ciencias Veterinarias y Pecuarias, Universidad de Chile, La Pintana 8820808, Santiago, Chile; LHerveClaude@rossvet.edu.kn (L.P.H.); christopher.hamilton@veterinaria.uchile.cl (C.H.-W.); 5 Biomedical Science Department, Ross University School of Veterinary Medicine, Basseterre KN269, Saint Kitts and Nevis; 6 Centro de Investigación Veterinaria Tandil, CONICET-CIC, Facultad de Ciencias Veterinarias, UNCPBA, Tandil 7000, Buenos Aires, Argentina; nlpadola@vet.unicen.edu.ar; 7 ANID—Millennium Science Initiative Program—Millennium Nucleus in the Biology of the Intestinal Microbiota, Macul 7830490, Santiago, Chile; 8 Joint Institute for Nutrition and Food Safety, University of Maryland, College Park, MD 20742, USA; jmeng@umd.edu; 9 Center for Food Safety and Applied Nutrition, U.S. Food and Drug Administration, College Park, MD 20740, USA; narjol.gonzalez-escalona@fda.hhs.gov

**Keywords:** STEC, non-O157, cattle, *stx* genes, virulence genes

## Abstract

**Simple Summary:**

Cattle are the main reservoir of Shiga toxin-producing *E. coli* (STEC), foodborne pathogens that cause severe disease and outbreaks. However, not all STEC cause human illnesses or have the same virulence potential. Characterizing strains isolated worldwide allows insights into how strains spread and which isolates have a more significant risk potential. This study described STEC isolation rates from cattle in Chile and characterized 30 isolates. We obtained 93 STEC isolates from 56/446 (12.6%) fecal cattle samples. Then, 30 non-O157 STEC isolates were selected for complete characterization; we found isolates of 16 different sequence types and 17 serotypes. One isolate was resistant to tetracycline and carried resistance genes against the drug. Surveyed virulence genes (*n* = 31) were present from 13% to 100% of isolates, and one isolate carried 26/31 virulence genes. Most isolates (90%; 27/30) carried the *stx2* gene, which is frequently linked to strains causing severe disease. A phylogenetic reconstruction demonstrated that isolates clustered based on serotypes, independent of their geographical origin (Central or Southern Chile). These results indicate that cattle in Chile carry a wide diversity of STEC potentially pathogenic for humans based on the presence of virulence genes.

**Abstract:**

Cattle are the main reservoir of Shiga toxin-producing *Escherichia coli* (STEC), one of the world’s most important foodborne pathogens. The pathogen causes severe human diseases and outbreaks. This study aimed to identify and characterize non-O157 STEC isolated from cattle feces from central and southern Chile. We analyzed 446 cattle fecal samples and isolated non-O157 STEC from 12.6% (56/446); a total of 93 different isolates were recovered. Most isolates displayed β-glucuronidase activity (96.8%; 90/93) and fermented sorbitol (86.0%; 80/93), whereas only 39.8% (37/93) were resistant to tellurite. A subgroup of 30 representative non-O157 STEC isolates was selected for whole-genome sequencing and bioinformatics analysis. In silico analysis showed that they grouped into 16 different sequence types and 17 serotypes; the serotypes most frequently identified were O116:H21 and O168:H8 (13% each). A single isolate of serotype O26:H11 was recovered. One isolate was resistant to tetracycline and carried resistance genes *tet(A)* and *tet(R)*; no other isolate displayed antimicrobial resistance or carried antimicrobial resistance genes. The intimin gene (*eae*) was identified in 13.3% (4/30) of the genomes and 90% (27/30) carried the *stx2* gene. A phylogenetic reconstruction demonstrated that the isolates clustered based on serotypes, independent of geographical origin. These results indicate that cattle in Chile carry a wide diversity of STEC potentially pathogenic for humans based on the presence of critical virulence genes.

## 1. Introduction

Cattle are the main reservoir of Shiga toxin-producing *Escherichia coli* (STEC) that can cause sporadic cases and foodborne outbreaks. Because illness can progress to severe complications such as hemolytic uremic syndrome (5–10% of cases), STEC is considered a public health concern [1,2]. While *E. coli* O157:H7 is the most notorious STEC serotype [3,4], over 400 non-O157 STEC have been identified, and over 100 of these have caused human disease worldwide [5,6]. Six STEC serogroups (O26, O45, O103, O111, O121, and O145) have been identified as the cause for most cases of non-O157 diseases in humans; the “Big Six” group [7]. Due to the public health impact of the Big Six, some countries, including the United States, have implemented policies banning the sale of some meat products found to carry these serogroups [8] and increased surveillance to detect them [5,8]. Laboratory surveillance is mandatory for STEC in Chile; the most frequently reported serotypes from 2011 to 2016 were O157, followed by O26 [9].

Animal colonization by STEC, especially carriage by domestic animals, can impact public health. Animal feces harboring STEC can spread through the environment and water sources contaminating produce, which has already been responsible for outbreaks of STEC-related illnesses among humans [10,11]. Cross-contamination can also occur during cattle slaughter or meat processing when good manufacturing practices are violated. This allows the meat to become contaminated with animal feces, tainting products and by-products with pathogens and finally causing human disease by ingesting these contaminated products [12,13]. Meat products have been the vehicle most frequently associated with STEC infections [6,14,15]. In Chile, previous studies have isolated STEC from ground beef [16]. In other countries, STEC has also been detected in pork and poultry processed under unsanitary conditions [17]. Also, direct contact with animals can result in human illness, as demonstrated by the number of outbreaks linked to petting zoos and animal fairs [14,18].

Understanding which strains are most likely to be pathogenic is of great importance to public health officials; therefore, molecular characterization of isolates to identify virulence factors is fundamental to understanding STEC infections’ epidemiology. Shiga toxins (Stx) are the characteristic virulence factor of STEC. Stx subtype 2 (encoded by the stx2 gene) has been more frequently linked to severe disease than Stx1 (encoded by stx1) [3]. Although isolates carrying stx can cause disease, the presence of these genes alone is not sufficient to cause human illness; numerous virulence factors have been described in STEC isolates [3]. Among the genes considered to be strongly associated with pathogenic isolates are adherence genes (e.g., *eae*, *iha*, *lpfA*), toxin genes (e.g., *stx*, *ehxA*, *subA*), and non-LEE encoded effectors (e.g., *nleA*, *nleB*, *nleC*, *efa1*), among others [3,19]. Therefore, molecular characterization of the isolates could help to predict their pathogenic potential. Likewise, the characterization of isolates obtained from different sources is fundamental to understand better the epidemiology of infection in diverse geographic areas, providing invaluable tools for public health officials [20].

Only limited data are currently available about which non-O157 STEC are present in cattle in Chile; major characteristics are unknown, including serotypes, sequence types, common virulence genes, and antimicrobial resistance genes. This study isolated, characterized, and sequenced the genome of non-O157 STEC isolates obtained from cattle raised in two geographic areas in Chile. This information will contribute to a better understanding of this pathogen’s diversity in cattle, the main STEC reservoir.

## 2. Materials and Methods

### 2.1. Sampling

We obtained 446 cattle fecal samples from dairy farms in two Chilean regions: 14 central (Region Libertador Bernardo O’Higgins; *n* = 155) and 5 southern (Region de Los Lagos and Region de Los Rios; *n* = 241) farms. We also included 1 southern slaughterhouse (*n* = 50) that processed meat and dairy animals (Supplementary Table S1). Samples were collected from November 2015 to December 2016. We recruited farms according to their interest in participating in the study, and oral consent was obtained from either the farm owner or slaughterhouse manager. At each site, trained veterinarians collected approximately 100 g of fecal samples per animal by manually massaging the animal’s rectum and placing the resulting material in a sterile bag or 150 mL sterile plastic container. Individual samples were kept below 8 °C during transportation to the Microbiology and Probiotics Laboratory at the University of Chile and then processed within 24 h.

### 2.2. PCR Screening and STEC Isolation

Fecal samples (*n* = 446) were individually enriched as previously described by Stromberg et al. (2015). Briefly, 1 g of fecal material was homogenized with 9 mL EC broth (BD Difco, Franklin Lakes, NJ, USA) and incubated at 37 °C for 18 h [21]. Then three loops of the enriched material were inoculated on McConkey agar (BD Difco) plates and incubated at 37 °C for 24 h. Bacterial DNA was extracted from the confluent growth area with the InstaGene^TM^ Matrix (Bio-Rad, Hercules, CA, USA) following manufacturer’s instructions. We ran a multiplex PCR screen to detect the *stx*_1_ and *stx*_2_ genes, using previously described conditions and primers [22] (Table 1). PCR reactions were performed using GoTaq^®^ Green Master Mix, following manufacturer’s instructions (Promega, Madison, WI, USA).

For each sample testing positive for *stx*_1_ and/or *stx*_2_, we re-isolated 30 colonies from the original McConkey plate where the bacterial DNA was previously extracted. These colonies were examined for the presence of *stx* genes by PCR, and *stx* positive isolates were later confirmed as *E. coli* by a PCR described by Chen et al. [24] (Table 1). We selected 1 to 4 STEC colonies from each positive sample and stored them in 20% glycerol at −80 °C until further analysis.

### 2.3. Preliminary Virulence Profiling and Molecular Serogrouping

Each of the STEC isolates obtained in the previous stage (*n* = 93) was analyzed for the presence of virulence genes *stx*_1_, *stx*_2_, *eae* and *hlyA* by PCR with previously described primers [22,24,25] using GoTaq^®^ Green Master mix (Promega, USA) and following manufacturer’s instructions (Table 1). A multiplex PCR was used to define whether the isolates belonged to one of the Big Six serogroups [22] (Table 1). STEC strain ATCC 35150 was used as a positive control for genes *stx*_1_, *stx*_2_, *eae* and *hlyA* reactions. Positive controls for the Big Six serogroups were DNA obtained from STEC isolates 88-353 (O26), A9619-C2 (O45), B27828/95 (O103), P1338 (O111), SJ18 (O121), and CVM9777 (O145) from the strain collection of the Food Safety Laboratory, University of Maryland [16].

### 2.4. Biochemical Characterization of STEC Isolates

Isolates in the study were additionally characterized for: (a) Sorbitol fermentation, by inoculating isolates on Sorbitol McConkey (SMAC) agar (BD, USA) [27]; (b) Tellurite resistance, by inoculating isolates on SMAC agar supplemented with 2.5 µg/mL potassium tellurite [27,28]; (c) β-glucuronidase activity, by inoculating isolates on TBX (Tryptone bile X-glucuronide) chromogenic agar (Biomérieux, Marcy-l’Étoile, France) [29]; and (d) Hemolysin production, by inoculating isolates on blood agar and washed sheep blood agar [28,30].

### 2.5. Selection of Non-O157 STEC for Full Characterization/WGS

We combined the above preliminary results to create bacterial profiles to select representative isolates; the goal was to examine isolates with diverse characteristics based on their phenotype (biochemical tests results) and genotype (as screened by PCR). First, we discarded isolates with the same profiles that were initially isolated from the same sample. Then, we selected representative isolates from Central and Southern Chile for further characterization (*n* = 30). Data used for selection are provided in Supplementary Table S2. From this point, all analyses were performed in this selected group of 30 non-O157 STEC isolates.

### 2.6. Saa Gene Typing in STEC Isolated from Cattle

We used a previously described PCR protocol to identify the presence of the gene *saa* and its variants in all 30 selected non-O157 STEC [31]. The primers used were VSAAF (5′-ACTCGCATAATTGGTGGTG-3′) and VSAAR (5′-ATCATTGGTATTGCTGTCAT-3′). This protocol identifies up to 6 *saa* variants depending on the amplicon size (6 variants from 0 to 5) [31]. GoTaq^®^ Green Master Mix was used for PCR reactions, following manufacturer’s instructions (Promega).

### 2.7. Antimicrobial Susceptibility

We determined the antimicrobial susceptibility of the 30 selected isolates to ampicillin (AMP) 10 µg, amoxicillin/clavulanic acid (AUX) 20/10 µg, ceftiofur (TIO) 30 µg, ceftriaxone (AXO) 30 µg, cefoxitin (FOX) 30 µg, gentamycin (GEN) 10 µg, streptomycin (STR) 10 µg, azithromycin (AZI) 15 µg, tetracycline (TET) 30 µg, ciprofloxacin (CIP) 5 µg, nalidixic acid (NAN) 30 µg, sulfisoxazole (FIS) 250 µg, trimethoprim/sulfamethoxazole (COT) 1.25/23.75 µg, and chloramphenicol (CHL) 30 µg with the disc diffusion technique (Oxoid, Hampshire, UK), following Clinical and Laboratory Standards Institute (CLSI) protocols [32]. The CLSI breakpoints were used for interpreting the inhibition halos [32]. Minimum inhibitory concentrations (MIC) were determined for any isolate that showed reduced susceptibility with the agar dilution technique [32].

### 2.8. Whole-Genome Sequencing (WGS)

Isolates selected for complete characterization (Table 2) were grown in TSB broth at 37 °C overnight. The DNeasy Blood and Tissue kit (Qiagen, Germantown, MD, USA) was used to extract DNA from the cultures. After measuring DNA concentration with a Qubit fluorimeter (Life Technologies, Carlsbad, CA, USA), the DNA concentration was standardized to 0.2 ng/µL. Libraries were prepared with the Nextera XT DNA kit (Illumina, San Diego, CA, USA), and genomes were sequenced using the Illumina MiSeq instrument (Illumina). The MiSeq Reagent Kit v2 of 500 cycles (2 × 250 pair-end reads) was used to sequence the isolates at the genomics laboratory of the US FDA Center for Food Safety and Applied Nutrition (CFSAN).

### 2.9. Genomic Data Analysis

De novo assemblies were crafted with the CLC Genomics Workbench Platform, Version 7.6.1 (Qiagen, USA), with default parameters and a minimum contig size of 500 bp. To characterize the isolates, we used the tools available at the website of the Center for Genomic Epidemiology (CGE), Technical University of Denmark (Lyngby, Denmark) (http://www.genomicepidemiology.org/, accessed on 19 April 2021). First, we determined each isolate’s sequence type using the MLST 1.8 tool [33]. This approach included the *E. coli* genes *adk*, *fumC*, *gyrB*, *icd*, *mdh*, *purA* and *recA*; allelic variations were used to assign numbers for sequence types (STs) [34]. We predicted the serotype for each draft genome using the SeroTypeFinder 1.1 tool [35] and the presence of virulence genes of *E. coli* using the VirulenceFinder 1.5 [36]. We also determined the *stx* and *eae* gene subtypes using a custom-made database in Ridom Seqsphere+ (Ridom GmbH, Germany) [37].

### 2.10. Phylogenetic Relatedness among Isolates

To analyze the phylogenetic relatedness among our isolates set, we called SNPs from the 30 assembled genomes using CSI phylogeny 1.4 (CGE) [38] with default parameters. *E. coli* K-12 MG1655 (GenBank Accession: NC_000913.3) was used as a reference genome. The resulting alignment was used to reconstruct the Maximum Likelihood (ML) phylogeny using RAxMLv.8 in GalaxyTrakr v8.2.4 (https://galaxytrakr.org/, accessed on 19 April 2021) with the GTR + CAT model to estimate genetic distances (1000 bootstrap re-sampling) [39]. The Newick file generated was visualized with Evolview [40].

We also performed a core genome MLST (cgMLST) analysis in Ridom Seqsphere+ software v4.1.9. We identified and defined target genes using *E. coli* K-12 MG1655 as the template genome. Then we compared the assembled genomes of our 30 STEC to the template, thereby defining the *core genome* of these STEC (Supplementary Table S3). Finally, a Minimum Spanning Tree (MST) was created to visualize the relationships among isolates. Clusters were defined as groups of genomes with fewer than 10 allele differences in their core gene content.

Additionally, a search for acquired antimicrobial resistance genes in the genomes was also performed, using the ResFinder 3.0 tool (CGE) [41,42].

### 2.11. Sequence Accession Numbers

All genomes (*n* = 30) were submitted to NCBI in SRA (Sequence Read Archive) format; accession numbers are in Table 2.

## 3. Results

In this study, we isolated non-O157 STEC from cattle from central and southern Chile and performed genotypic, phenotypic, and bioinformatics characterization of the isolates.

### 3.1. STEC Detection and Preliminary Characterization by PCR

**Figure 1:**
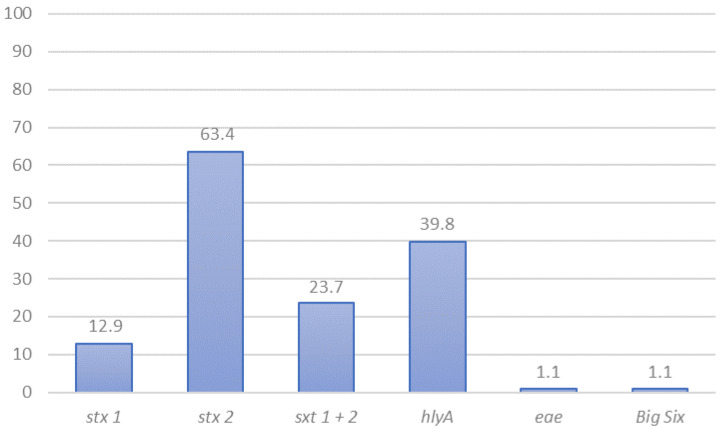
Genotypic characterization of non-O157 STEC isolated from cattle in Chile (*n* = 93) by the presence of virulence genes (%). Genes were detected by PCR as described in references [22,23,25,26].

Overall, PCR screening for genes *stx*_1_ and/or *stx*_2_ resulted in 44.6% (208/446) samples with STEC presumptive presence; 55.5% (86/155) from central Chile, and 41.9% (122/291) for southern Chile. STEC isolation rates from fecal samples were lower (Table 3). Of the 56 samples where we isolated STEC, one to four isolates were further investigated, and a total of 93 different isolates were tested for the presence of the most common STEC virulence genes. Isolates with the same characteristics coming from the same samples were eliminated from the analysis. *stx*_1_ was carried as the only Shiga toxin gene by 12.9% of isolates (12/93), *stx*_2_ by 63.4% (59/93), and the combination *stx*_1_ + *stx*_2_ was present in 23.7% (22/93) of isolates (Figure 1). A single isolate carried *eae* as detected by PCR, while *hlyA* was detected in 39.8% (37/93) of isolates (Figure 1). One isolate was serogroup O26, and it was the only isolate belonging to the “Big Six” group (Supplementary Table S2).

### 3.2. Phenotypic Characteristics of STEC Isolated from Cattle

**Figure 2:**
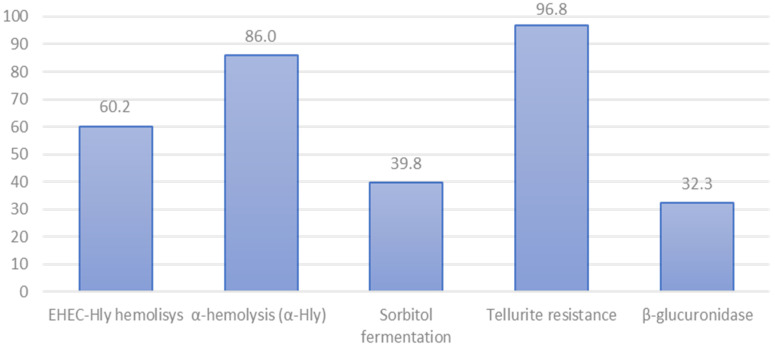
Phenotypic characterization of non-O157 STEC isolated from cattle in Chile (*n* = 93) in percentages (%).

Phenotypic characterization showed that 96.8% (90/93) of our isolates were β-glucuronidase positive, 86.0% (80/93) fermented sorbitol, 18.3% (17/93) produced EHEC-*hly* hemolysis, and 60.2% (56/93) produced α-hemolysin (Figure 2). Also, 39.8% (37/93) were resistant to tellurite at the tested concentration. Interestingly, 22.9% (11/48) of the central Chile isolates were resistant to tellurite, while over 57.8% (26/45) of the isolates from southern Chile were resistant to tellurite (Supplementary Table S2).

### 3.3. Antimicrobial Susceptibility

A subgroup of 30 isolates was chosen for further characterization (Table 2). Selected isolates were analyzed for antimicrobial susceptibility using the disc diffusion method against 14 antimicrobial agents. Only one isolate, E6-III, displayed reduced susceptibility to tetracycline (TET). We determined the minimum inhibitory concentration to TET to confirm this finding, which showed that E6-III was highly resistant to tetracycline (MIC > 64 µg/mL) [32]. This phenotype was consistent with the results obtained from the genomic search of antimicrobial resistance genes (ResFinder), whose results showed that the only antimicrobial isolate with resistance genes detected was E6-III, which carried genes *tet(A)* and *tet(R)* located next to each other. No other AMR gene was found in the E6-III genome or any other STEC.

### 3.4. Sequence Types and Serotypes of Sequenced STEC by Genomic Analyses

WGS was conducted in those 30 isolates chosen for further characterization (Table 2). STEC sequenced were of 16 different Sequence Types (ST), and 17 different serotypes were predicted from their genomic sequences (Table 2). Seven isolates belonged to Clonal Complex (CC) ST155, and three isolates belonged to CC ST205. Among the most frequent serotypes, serotypes O116:H21 and O168:H8 were each represented by four genomes. Four genomes were identified as O153/O178:H18 (Table 2). All serotypes were generally found in both central and southern Chile (Table 2).

### 3.5. Virulence Genes in STEC Isolated in Chile

**Figure 3:**
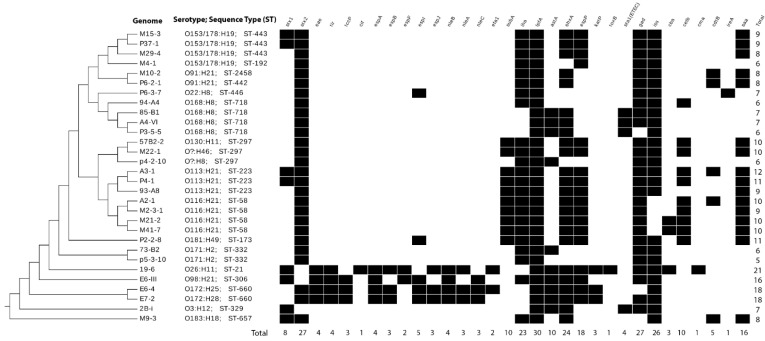
Matrix of the presence/absence of virulence genes in STEC genomes isolated from cattle in Chile (*n* = 30). Black filling indicates that the gene is present. All genes but *saa* were surveyed in each genome using the VirulenceFinder software from DTU [36]. Gene *saa* was screened through PCR and confirmed by in silico PCR from their WGS and primers described in Lucchesi et al., 2006 [31].

Using VirulenceFinder (CGE) [36], we identified 31 virulence genes among the 30 isolates analyzed. These genes were detected in different frequencies and combinations (Figure 3), ranging from 5 to 21 virulence genes per genome. The most frequently found gene was *lpf*A (long polar fimbriae A; adhesin), present in all 30 genomes sequenced. Genes *gad* and *stx*_2_ were detected in 90% (27/30) of the genomes. In contrast, gene *stx*_1_ was present in only 26.7% (8/30) of the sequenced genomes, and *eae* was identified in only 13% (4/30) of them (Figure 2).

Genes closely linked to *eae* (*tir*, *espA*, *espB*, espJ, *nleA*, *nleB* and *nleC*) were also detected in 10% to 13% of isolates; those isolates also carried *eae*. Genes *toxB* (toxin B; adhesin), *cma* (colicin-M; toxin), and *cif* (cycle inhibiting factor; non-LEE effector) were detected in a single isolate (isolate 19-6; O26:H11); that same isolate carried 21 virulence factors, which was the highest number found in a single isolate. Genomes within serogroup O172 (*n* = 2: H25 and H28) carried 18 virulence genes, including *eae*, and one serotype O98:H21 isolate displayed 16 virulence genes. Isolate p5-3-10 (O171:H2) carried five virulence genes, which was the lowest number found for an isolate in this study (Figure 3).

The *saa* gene was detected in 16/30 STEC (53.3%) isolates, and four *saa* variants were detected (1, 2, 3 and 4). All those genomes also carried genes *ehxA* and *stx*_2_ (Figure 3). Variant 1 was the most frequently *saa* variant identified (6/16; 37.5%), and a single isolate carried variant 4 (M22-1) (Figure 3; Supplementary Table S2).

### 3.6. Phylogenetic Relatedness among Genomes

**Figure 4:**
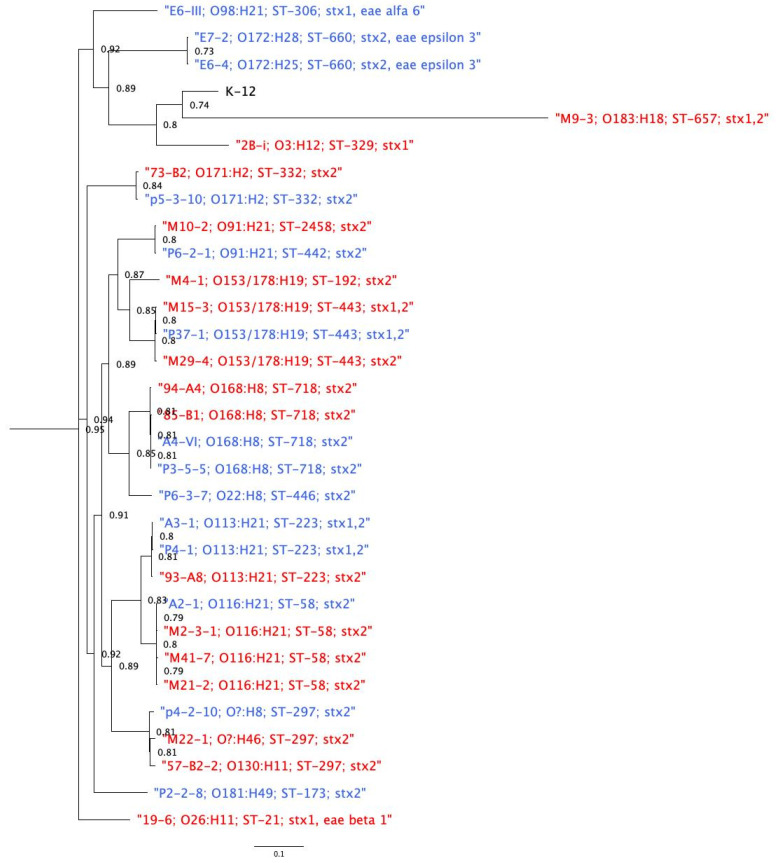
Maximum likelihood phylogeny of STEC isolated from cattle in Chile (*n* = 30). The evolutionary history was inferred using the maximum likelihood method using RAxML with SNPs inferred from CSI phylogeny. The tree is drawn to scale, with branch lengths measured in the number of substitutions per site. Bootstrap values are shown. Isolates in red represent southern isolates, and blue represents central isolates. SNP matrix is available in Table S4.

The SNP matrix resulted in 83,361 SNPs. Using these to construct the Maximum Likelihood (ML) phylogeny (Figure 4), the analysis showed that isolates of the same sequence type and serotype clustered together. The geographic location where the isolates were obtained did not influence clustering. However, four isolates exhibited unusual patterns: isolates M10-2 and P6-2-1 were of different ST (ST2458 and ST442) but shared the same serotype (O91:H21) and formed a cluster; isolates E7-2 and E6-4 were of different serotypes (O172:H28 and O172:H25) but showed the same ST (ST660), also forming a cluster (Figure 4).

Further confirmation of our phylogenetic findings came from running a core genome MLST (cgMLST) study. We identified 3925 target genes in the genome of *E. coli* K-12 (GenBank Accession: NC_000913.3), of which 2351 genes represented the core STEC genome used to study relatedness among isolates. A Minimum Spanning Tree (MST) showed that our isolates formed three clusters, which grouped genomes having the same ST and serogroup. Interestingly, two genomes formed one cluster with the same O type but different H type (Cluster 1; Supplementary Figure S1). We found a range of 5–153 differences in the core genes among the serotypes identified. Isolates obtained from different geographic areas but sharing the same serotype showed as few as 32 different core genes (Supplementary Figure S1).

## 4. Discussion

In the current study, we isolated and characterized non-O157 STEC obtained from cattle in two locations in Chile. Different serotypes were identified, and we observed diverse combinations of virulence factors among the isolates; a high percentage of isolates carried high-risk virulence gene combinations, such as *stx2/saa/exhA*.

We isolated STEC from 12.5% of the cattle fecal samples analyzed; preliminary genotypic characterization of the 93 different isolates showed that *stx*_2_ was the most frequent Shiga toxin gene variant among the isolates. Several studies have demonstrated that Stx_2_ is more frequently linked to severe hemorrhagic colitis and HUS cases than Stx_1_ [43,44]. Our results differ from a study carried out in 1989 in Chile [45], which identified *stx*_1_ as the most frequently Shiga toxin gene detected in STEC isolated from cattle from a slaughterhouse. Divergent results between both Chilean studies might be due to two main factors: (i) Most of our samples came from dairy instead of beef cattle, and (ii) Samples were taken 30 years apart, hinting at a possible epidemiological shift. Supporting this, *stx*_2_ was the most frequently detected Shiga toxin gene in a recent study investigating STEC isolated from ground beef from Santiago, Chile [16], and similar Shiga toxin gene frequencies were described in STEC from cattle in Argentina, our neighboring country [46]. However, more studies are necessary to confirm this hypothesis, mainly because this study was not designed as an epidemiological study and samples were taken by convenience, and a structured sample was not used. We also detected different STEC isolation percentages between central and southern Chile. Even when all farms surveyed were dairy farms, differences in farm sizes (Central Chile farms held <20 animals versus 200 to 500 animals in Southern farms), animal density, geographical zones, and weather might result in different management practices that could influence STEC carriage [46,47], however, this study was not designed to determine the prevalence of STEC in Chile or differences between areas, and further studies are necessary.

We identified 16 ST and 17 serotypes in the 30 sequenced genomes, demonstrating a wide diversity among isolates (Supplementary Table S2). Serotypes O116:H21 ST58 and O168:H8 ST718 were the most represented; these have been previously isolated from cattle [48,49,50]. Furthermore, serotypes O26:H11, O91:H21, O113:H21, O116:H21, and O130:H11, characterized in this study, have also caused disease and human outbreaks [51,52,53]. For example, STEC O116:H21 was isolated in Argentina from ground beef [54] and cattle feces [55]. In Paraguay, O116:H21 was isolated from cattle [56]. STEC O113:H21 has been isolated in the US and Canada from cattle, pigs, and water obtained in the surroundings of cattle farms [57]. The latter has caused HUS in both countries [58,59] and in Australia in 1998 [23]. In Chile, official reports indicate STEC O113 is one of the most frequent serogroups isolated from meats [9]. In Argentina, STEC O113 has been isolated from ground beef and cattle, and it has also caused human disease, rising as an emerging serotype in the country [53,54]. We also reported one strain of the Big Six groups: STEC O26:H11. The “Big Six” group (serogroups O26, O45, O103, O111, O121, and O145) have epidemiological importance in the US and around the world since they have caused multiple foodborne outbreaks [60,61]. Moreover, the Chilean Public Health Institute informed that serotypes O157:H7 and O26:H11 are the most frequently reported causes of disease by STEC in Chile. Altogether, this information shows that some of the bovine isolates obtained in this study might represent a risk for public health.

We detected various frequencies and combinations of 32 virulence genes (including *saa*). Four of the genomes in this study carried genes *eae* and *tir*; most LEE effector genes present in LEE positive isolates are required for close adherence and form the effacing and attaching lesion [62], moreover, the simultaneous presence of *stx*_2_, *eae*, *exhA* and has been frequently linked to severe disease. This combination was found in two O172 strains. Additionally, O26:H11 is the non-O157 STEC serotype most frequently causing human disease [9,37,51], also indicating a potentially high risk for the population. In this collection, we found virulence gene *lpf*A (long polar fimbria) in every genome in the study. The protein that *lpf*A encodes, Lpf, contributes to *E. coli* to adhere to human intestinal cells, and it has recently been associated with a pro-inflammatory response to infection [63,64]. Among the LEE-negative genomes, we found virulence genes such as *saa*, *iha* and *subA*, and we also found genes *toxB* and *efa*1, both frequently found in LEE-negative isolates [19,65]. *saa* is one of the most important virulence genes in non-LEE isolates; it has been associated with developing severe disease in humans [19,66]. Due to variations in copy number—this gene has a repetition sequence of 111 bp—the size *of saa* can also vary; therefore, *saa* is not included as a target gene in the bioinformatics software VirulenceFinder 1.5 (CGE) [19,31]. In this study, 16/30 (53%) genomes carried the *stx*_2_, *saa*, *exh*A combination, which is also considered high-risk [67]. Interestingly, we found isolates of the same serotype and ST with different virulence profiles, highlighting that those isolates obtained from cattle are diverse.

Antimicrobial resistance among STEC is not frequent, but an increase in resistance has been recently described [68]. We found only one isolate that displayed AMR, which was resistant to tetracycline. This antimicrobial is used in animal and human health; studies have found that up to 100% of STEC isolated from humans and cattle was resistant to this drug [69]. Antimicrobial resistance genes *tet(A)* and *tet(R)* found in this study have been reported in STEC and also in other Enterobacteriaceae [70,71,72].

The phylogenetic reconstruction showed a wide diversity of STEC isolated from cattle in Chile. As expected, most of these isolates clustered based on ST and serotype, and it appears that the geographic origin of an isolate had only a marginal influence on clustering. While we expected that isolates within the same ST and from the same geographical area would cluster together, we did identify a case where one isolate from southern Chile clustered with two isolates from central Chile, without clustering with another southern Chile isolate of the same serotype (Figure 1; Supplementary Figure S1) Although this connection is intriguing, we did not have background information about the specific animals surveyed which might have helped to explain the phenomenon. Other studies are required to discover the extent of this finding, perhaps by investigating cattle transport between areas or other factors that influence the survival of particular strains in specific settings.

We found several genomes potentially carrying mobile genetic elements. For example, the gene *saa* has been described as part of the pO113 plasmid, especially in STEC serotype O113:H21 LEE-negative [66,73], and it has also been reported in STEC O91:H21 and O20:H19 isolated from cattle feces and beef hamburgers [74]. However, *saa* has also been reported in STEC O113:H21 that lack pO113 but carry the LAA pathogenicity island (Locus of Adhesion and Auto-aggregation) [74]. The presence of mobile genetic elements, such as plasmids and pathogenicity islands, could play a role in phylogenetic reconstructions; however, these elements are not considered in phylogeny when using a reference genome or in cgMLST as in the current study, since they are not part of the core genome.

The results we reported may have underestimated the actual prevalence of STEC present in cattle in Chile; we were only able to isolate STEC from 25% of positive samples for *stx* genes at the screening. Our approach for isolating these STEC first used a high sensitivity method (a PCR screening), followed by one with lower sensitivity (culture and isolation of 30 colonies in plates) [75]. Similar challenges have been reported in previous studies [76,77]. Surveying more colonies per sample could increase the isolation rate; the ISO protocol recommends testing 30 to 200 colonies for samples that tested positive to the screening. Moreover, using a different methodological approach, such as immunomagnetic concentration or chromogenic agars, could reveal additional STEC. Moreover, *stx* genes can be found in other *E. coli* (EPEC) and other bacterial species such as *Citrobacter freundii*, *Shigella* spp., among others which might explain part of these results [78,79]. However, it is important to clarify that this study was not designed to determine the actual non-O157 STEC prevalence in Chile; this study is a first approximation in assessing the problem of STEC in cattle in Chile, and further studies are necessary. Genome serotype prediction did not allow differentiation between isolates of serogroups O153 and O178. This happens because the sequences of genes *wzx* and *wzy* are used to define O types in STEC. However, these sequences are identical in O153 and O178, and therefore we could not identify the serogroup of these four isolates [35]. Finally, we were surprised to find that although four genomes carried *eae*, the PCR screening detected only one non-O157 STEC carrying the *eae* gene. To understand the problem better, we performed in silico PCR with the primers used for screening [25]. The results indicated that the primers used misaligned *eae* genomes in our collection (data not shown), so we decided to use a different set of primers in future studies.

## 5. Conclusions

Our results provide insights into the large diversity of STEC isolated from cattle in two Chilean locations. Considering that some of the serotypes were found in combination with virulence factors in the isolates, cattle may be a source of potential pathogenic STEC in Chile. Therefore, some of the food products derived from these colonized animals (such as milk, cheese, and meat) could become contaminated with potentially pathogenic STEC, risking public health. Farmworkers and their families may also be at risk due to close contact with farm animals and animal feces. Consequently, control measures must be enforced at different food production levels to avoid STEC spread to humans, foods, and animals. Specifically, improving good manufacturing practices would help avoid the contact of different food products with animal feces that can carry these human pathogens and become a vehicle of foodborne diseases.

## Figures and Tables

**Table 1:** Primers used for detection and characterization of STEC isolates.

Type of PCR	Target	Forward Primer (5′ to 3′)	Reverse Primer (5′ to 3′)	Annealing Temp (°C)	Amplicon Size (bp)	Reference
Multiplex or Singleplex	*stx*_1_	CAGTTAATGTGGTGGCGAAGG	CACCAGACAATGTAACCGCTG	56	348	[22,23]
	*stx*_2_	ATCCTATTCCCGGGAGTTTACG	GCGTCATCGTATACACAGGAGC		584	
Singleplex	*E. coli uspA*	CCGATACGCTGCCAATCAGT	ACGCAGACCGTAGGCCAGAT	58	884	[24]
Singleplex	*eae*	ATTACCATCCACACAGACGGT	ACAGCGTGGTTGGATCAACCT	63	397	[25]
Singleplex	*hlyA*	AGCCGGAACAGTTCTCTCAG	CCAGCATAACAGCCGATGT	60	526	[26]
Multiplex or Singleplex	O26 *wzx*	GTGTGTCTGGTTCGTATTTTTTATCTG	CCTTATATCCCAATATAGTACCCACCC	56	438	[22]
	O45 *wzx*	GGTCGATAACTGGTATGCAATATG	CTAGGCAGAAAGCTATCAACCAC		341	
	O103 *wzy*	TTATACAAATGGCGTGGATTGGAG	TGCAGACACATGAAAAGTTGATGC		385	
	O111 *wzx*	TTCGATGTTGCGAGGAATAATTC	GTGAGAGCCCACCAGTTAATTGAAG		362	
	O121 *wzy*	AGTGGGGAAGGGCGTTACTTATC	CAATGAGTGCAGGCAAAATGGAG		366	
	O145 *wzy*	CCTGTCTGTTGCTTCAGCCCTTT	CTGTGCGCGAACCACTGCTAAT		392	

**Table 2:** Genetic characteristics of sequenced STEC (30) isolated in Chile.

Isolate Name	CFSAN Number	Accession Number (SRA)	Location *	Sequence Type	Serotype	*stx* Gene Subtype	*eae* Gene Subtype
M22-1	CFSAN066373	SRX3735307	Southern	297	O?:H46	2	-
p4-2-10	CFSAN066356	SRX3735281	Central	297	O?:H8	2	-
A3-1	CFSAN066341	SRX3735341	Central	223	O113:H21	1, 2	-
93-A8	CFSAN066380	SRX3735290	Southern	223	O113:H21	2a	-
P4-1.	CFSAN066382	SRX3735276	Central	223	O113:H21	1a, 2a	-
A2-1	CFSAN066340	SRX3735340	Central	58	O116:H21	2a	-
M21-2	CFSAN066372	SRX3735329	Southern	58	O116:H21	2	-
M2-3-1	CFSAN066391	SRX3735309	Southern	58	O116:H21	2a	-
M41-7	CFSAN066376	SRX3735286	Southern	58	O116:H21	2	-
57-B2-2	CFSAN066390	SRX3735310	Southern	297	O130:H11	2	-
M4-1	CFSAN066365	SRX3735322	Southern	192	O153/178:H19	2	-
M15-3	CFSAN066370	SRX3735321	Southern	443	O153/178:H19	1, 2	-
M29-4	CFSAN066375	SRX3735287	Southern	443	O153/178:H19	2c	-
P37-1	CFSAN066386	SRX3735308	Central	443	O153/178:H19	1a, 2a	-
A4-VI	CFSAN066342	SRX3735338	Central	718	O168:H8	2g	-
P3-5-5	CFSAN066355	SRX3735282	Central	718	O168:H8	2g	-
85-B1	CFSAN066379	SRX3735291	Southern	718	O168:H8	2g	-
94-A4	CFSAN066381	SRX3735277	Southern	718	O168:H8	2	-
p5-3-10	CFSAN066357	SRX3735280	Central	332	O171:H2	2a	-
73-B2	CFSAN066378	SRX3735288	Southern	332	O171:H2	2c	-
E6-4	CFSAN066346	SRX3735347	Central	660	O172:H25	2a	Epsilon-3
E7-2	CFSAN066349	SRX3735285	Central	660	O172:H28	2a	Epsilon-3
P2-2-8	CFSAN066354	SRX3735283	Central	173	O181:H49	2c	-
M9-3	CFSAN066366	SRX3735323	Southern	657	O183:H18	1, 2	-
P6-3-7	CFSAN066360	SRX3735293	Central	446	O22:H8	2c	-
19-6	CFSAN066388	SRX3735346	Southern	21	O26:H11	1a	Beta-1
2B-i	CFSAN066353	SRX3735284	Southern	329	O3:H12	1a	-
M10-2	CFSAN066367	SRX3735324	Southern	2458	O91:H21	2a	-
P6-2-1	CFSAN066358	SRX3735279	Central	442	O91:H21	2a	-
E6-III	CFSAN066345	SRX3735339	Central	306	O98:H21	1a	Alpha-6

Sequence type, serotype, *stx* gene subtype, and *eae* gene subtype were predicted using whole-genome sequence data. * Central Chile samples were taken in Región del Libertador Bernardo O’Higgins while southern Chile samples were obtained in the Región de Los Lagos and Región de Los Ríos.

**Table 3:** STEC screening and isolation rates in cattle from central and southern farms in Chile.

Location	Number of Farms	Isolation (%)
Central	14	39/155 (25.2)
Southern	5 + 1 slaughterhouse	17/291 (5.8)
Total	20	56/446 (12.5)

Central Chile samples were obtained in Región del Libertador Bernardo O’Higgins while southern Chile samples were obtained in the Región de Los Lagos and Región de Los Ríos.

## Data Availability

All genomic data are available at NCBI. Please refer to Table 2 for details.

## Supplementary Materials

The following are available online at https://www.mdpi.com/article/10.3390/ani11082388/s1, Figure S1: Minimum Spanning Tree (MST) of STEC genomes isolated from cattle (*n* = 30), Table S1: Percentage of STEC detected by farm in the study, Table S2: Preliminary genotypes and phenotypes of 91 non-O157 STEC isolates from Bovine. Table S3: Allele Matrix generated by a cgMLST analysis from 30 STEC genomes. Table S4: SNP distance Matrix of STEC genomes.
