# Supplementary material for: Diversity of Non-O157 Shiga Toxin-Producing Escherichia coli Isolated from Cattle from Central and Southern Chile
Source: Animals (Basel). 2021 Aug 13;11(8):2388. doi: 10.3390/ani11082388 (PMC8388633; doi:10.3390/ani11082388)
Supplement: Supplementary file 1 [file animals-11-02388-s001.zip › Figure S1.pdf]

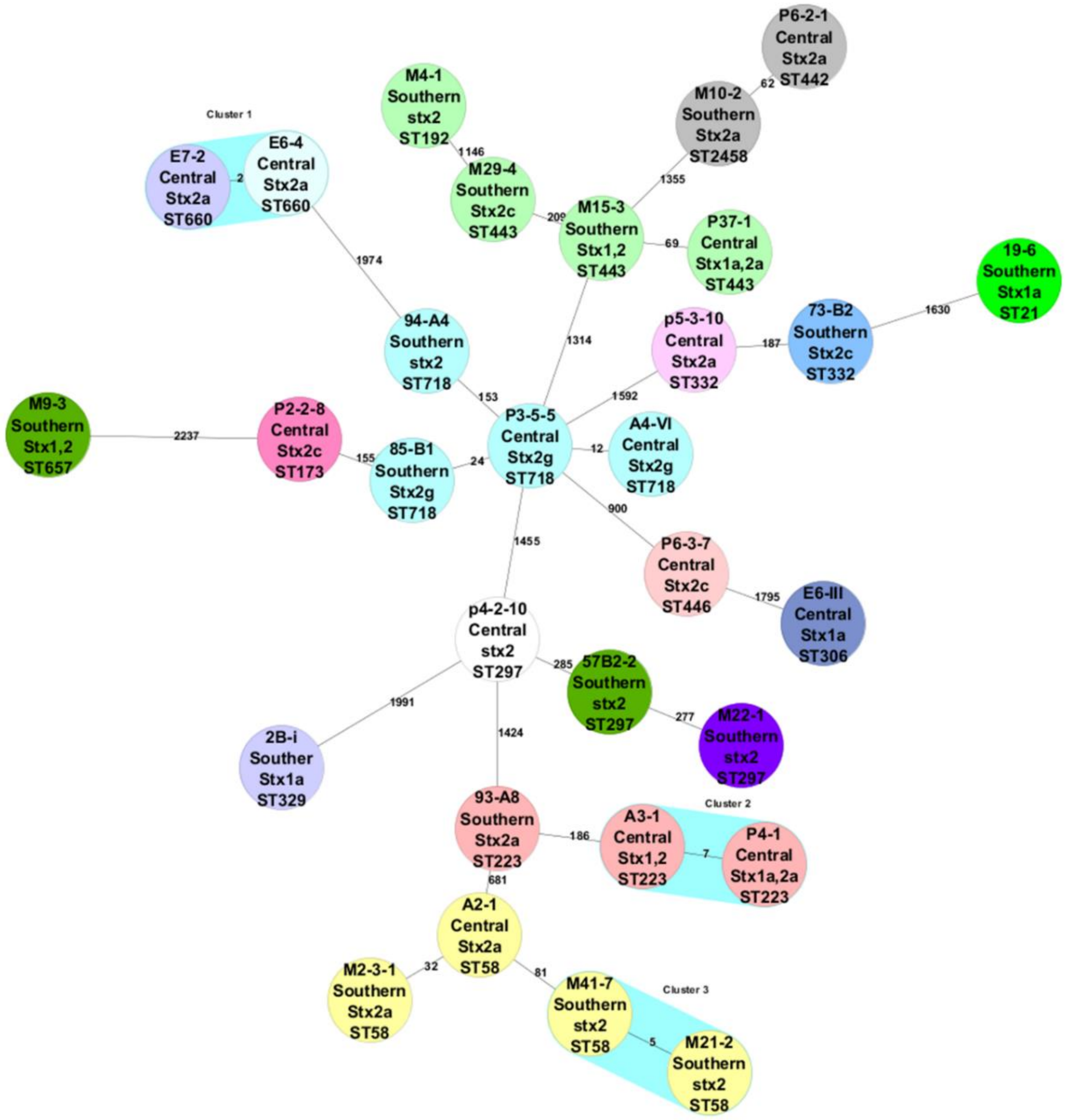

Supplementary Figure S1. Minimum Spanning Tree (MST) of STEC genomes isolated from cattle (*n* = 30). Each circle represents an isolate. Each color indicates a serotype. Numbers on lines between circles indicate allele differences among the genomes. Each circle contains the isolate identification code, the origin of the isolates (central or southern Chile), *stx* type, the sequence type (ST). Three clusters are represented by a light blue area merging two or more circles and were defined as genomes with fewer than ten allele differences. A total of 2351 genes representing the STEC core genome was used to construct the MST.
